# Predicting the development of normal tension glaucoma and related risk factors in normal tension glaucoma suspects

**DOI:** 10.1038/s41598-021-95984-7

**Published:** 2021-08-17

**Authors:** Hae-Young Lopilly Park, Da Young Shin, Soo Ji Jeon, Yong-Chan Kim, Younhea Jung, Eun Kyoung Kim, Hye-Young Shin, Kyoung In Jung, Jin A. Choi, Na Young Lee, Seung Woo Hong, Chan Kee Park

**Affiliations:** 1 Department of Ophthalmology, The Catholic University of Korea, Seoul, Korea; 2 Seoul St. Mary’s Hospital, Seoul, Korea; 3 Incheon St. Mary’s Hospital, Incheon, Korea; 4 Yeouido St. Mary’s Hospital, Seoul, Korea; 5 Uijeongbu St. Mary’s Hospital, Uijeongbu, Korea; 6 St. Vincent’s Hospital, Suwon, Korea; 7 Eunpyeong St. Mary’s Hospital, Seoul, Korea; 8 Bucheon St. Mary’s Hospital, Bucheon, Korea

**Keywords:** Diseases, Medical research, Risk factors

## Abstract

This study investigated the predicted risk factors for the development of normal-tension glaucoma (NTG) in NTG suspects. A total of 684 eyes of 379 NTG suspects who were followed-up for at least 5 years were included in the study. NTG suspects were those having (1) intraocular pressure within normal range, (2) suspicious optic disc (neuroretinal rim thinning) or enlarged cup-to-disc ratio (≥ 0.6), but without definite localized retinal nerve fiber layer (RNFL) defects on red-free disc/fundus photographs, and (3) normal visual field (VF). Demographic, systemic, and ocular characteristics were determined at the time of the first visit via detailed history-taking and examination of past medical records. Various ocular parameters were assess using spectral-domain optical coherence tomography and Heidelberg retinal tomography. Conversion to NTG was defined either by the presence of a new localized RNFL defect at the superotemporal or inferotemporal region on disc/fundus red-free photographs, or presence of a glaucomatous VF defect on pattern standard deviation plots on two consecutive tests. Hazard ratios were calculated with the Cox proportional hazard model. In total, 86 (12.6%) of the 684 NTG suspects converted to NTG during the follow-up period of 69.39 ± 7.77 months. Significant (*P* < 0.05, Cox regression) risk factors included medication for systemic hypertension, longer axial length, worse baseline VF parameters, thinner baseline peripapillary RNFL, greater disc torsion, and lamina cribrosa (LC) thickness < 180.5 μm (using a cut-off value obtained by regression analysis). Significant (*P* < 0.05, Cox regression) risk factors in the non-myopic NTG suspects included medication for systemic hypertension and a LC thinner than the cut-off value. Significant (*P* < 0.05, Cox regression) risk factors in the myopic NTG suspects included greater disc torsion. The results indicated that 12.6% of NTG suspects converted to NTG during the 5–6-year follow-up period. NTG suspects taking medication for systemic hypertension, disc torsion of the optic disc in the inferotemporal direction, and thinner LC of the optic nerve head at baseline were at greater risk of NTG conversion. Related baseline risk factors were different between myopic and non-myopic NTG suspects.

## Introduction

Glaucoma suspects are defined as individuals with clinical findings or risk factors that may increase the likelihood of developing glaucoma, including high intraocular pressure (IOP), suspicious appearance of the optic disc or the retinal nerve fiber layer (RNFL), and occludable or narrow angles in the anterior chamber. However, the conversion rate of glaucoma suspects to glaucoma varies widely according to the clinical findings and risk factors. In addition, there have been few large longitudinal studies to identify clinical factors associated with higher risk of glaucoma development in glaucoma suspects. The Ocular Hypertension Treatment Study (OHTS), a randomized clinical study of glaucoma suspects with high IOP, showed that treatment reduced the conversion rate of glaucoma suspects to glaucoma. This study also provided a risk calculator allowing ocular hypertension (OHT) subjects to predict their risk of developing glaucoma. However, the study included only individuals without a suspicious appearance of the optic disc.

Most of the suspected individuals of glaucoma initiate evaluation of glaucoma and routine follow-up when high IOP or suspected findings are found in their optic disc and/or RNFL during ocular examination or glaucoma screening. There have been limited reports related to the risk factors of glaucoma suspects with IOP within the normal range, but with suspicious optic disc or RNFL findings, which we call normal-tension glaucoma (NTG) suspects who can develop NTG. The risk factors to predict glaucoma development from OHTS could not be applied to NTG suspects. In addition, it is now possible to obtain more data on glaucoma suspects compared to when the OHTS was conducted, by using spectral-domain optical coherence tomography (SD-OCT) to analyze the optic nerve head (ONH) or gathering information regarding the ganglion cell/inner plexiform layer (GC/IPL) thickness in the macula region. Therefore, we need a comprehensive analysis of NTG suspects and find out their risk and related risk factors for developing NTG to manage these individuals.

Less than 10% of glaucoma suspects developed glaucoma during the 5-year follow-up period in OHTS^1–3^. The rate of developing glaucomatous visual field (VF) damage in individuals with suspicious optic disc or RNFL, but with normal VF and IOP within the normal range (mostly classified as normotensive preperimetric glaucoma), was reported to range from 13% to 57.7% after a 3–5-year follow-up period^4–8^. This suggests wide variation in the likelihood of developing glaucoma according to the possessed risk factors. Identifying prognostic risk factors for the development of NTG is therefore important, because they may help to determine whether medication is required and allow the follow-up intervals to be customized during management of NTG suspects. Myopia is a well-known risk factor for glaucoma and we previously found that up to 73.8% of NTG patients had myopia^9,10^. Therefore, looking into the difference between myopic and non-myopic NTG suspects and comparing the related risk factors for glaucoma development may help us managing these NTG suspects. Here, we performed a multicenter longitudinal observational study of NTG suspects to identify risk factors for developing NTG based on demographic, systemic, and ocular characteristics, structural and functional ocular parameters, and measurements of the optic disc and ONH parameters performed using current diagnostic techniques. Additionally, analysis was performed to find out risk factors for NTG development in NTG suspect according to the presence of myopia.

## Results

A total of 734 eyes of 411 NTG suspects satisfied the inclusion and exclusion criteria. Of these 734 eyes, 50 (6.8%) were excluded from the analysis because the disc/RNFL photographs or OCT images were of poor quality, or the VF reliability indices were unreliable. Therefore, a total of 684 eyes of 379 NTG suspects were analyzed in this study. The ONH measurements showed excellent reproducibility, with ICCs for ONH measurements of 0.985 (95% CI = 0.974–0.995) for prelaminar thickness, 0.974 (95% CI = 0.961–0.982) for LC depth-BMO (Bruch’s membrane opening), 0.982 (95% CI = 0.965–0.989) for LC depth-PPS (peripapillary sclera), and 0.952 (95% CI = 0.931–0.973) for LC thickness.

The baseline characteristics of the NTG suspects are listed in Table 1. The mean age of the patients was 52.11 ± 15.96 years. There was a family history of glaucoma in 76 (11.1%) subjects. In total, 42 (6.1%) subjects were taking medication for diabetes mellitus and 111 (16.2%) for systemic hypertension. The mean follow-up duration was 69.39 ± 7.77 months. Of the 684 NTG suspect eyes, 86 (12.6%) progressed to NTG (Table 2). Among these progressed eyes, 20 (23.3%) were found on two consecutive VF tests, 36 (41.9%) on RNFL photography, and 30 (34.9%) on both tests. There was 8 (9.3%) who underwent cataract surgery in the progressor group and 79 (13.2%) in the non-progressor group (*P* = 0.388). Subjects with eyes that converted to NTG were significantly older (*P* = 0.019), had a higher frequency of family history of glaucoma (*P* = 0.027), were more likely to be taking medication for systemic hypertension (*P* < 0.001), and had eyes with a longer axial length (*P* = 0.031) than those who did not convert to NTG. The baseline average peripapillary RNFL thickness (*P* < 0.001) and average macular GC/IPL thickness (*P* = 0.017) were thinner in NTG suspect eyes that converted to NTG than in eyes that did not convert to NTG. The baseline PSD of the VF (*P* < 0.001) was worse, and disc hemorrhage (*P* = 0.018) was more frequent during the follow-up period in NTG suspect eyes that converted to NTG. Measurement of posterior profiles and ONH parameters showed that NTG suspect eyes that converted to glaucoma had greater disc torsion in the inferotemporal direction (*P* = 0.016) and a significantly larger PPA area (*P* = 0.015).

**Table 1:** Baseline demographics and ocular characteristics of 684 eyes of 379 NTG suspects.

Variables	Description
**Demographics**	
Age at diagnosis (y)	52.11 ± 15.96
Female, no. (%)	444 (64.9)
Family history of glaucoma, no. (%)	76 (11.1)
**Systemic demographics**	
Medication of DM, no. (%)	42 (6.1)
Medication of HTN, no. (%)	111 (16.2)
**Ocular demographics**	
Best corrected visual acuity	0.93 ± 0.12
Axial length (mm)	24.49 ± 1.99
Central corneal thickness (μm)	546.59 ± 44.88
**IOP parameters**	
Baseline IOP (mmHg)	15.99 ± 3.68
Mean follow-up IOP (mmHg)	15.33 ± 3.48
**OCT parameters**	
Baseline average pRNFL thickness (μm)	91.21 ± 9.53
Baseline average mGC/IPL thickness (μm)	78.43 ± 9.86
**VF parameters**	
Baseline MD of SAP (dB)	− 1.30 ± 1.83
Baseline PSD of SAP (dB)	1.83 ± 0.85
**Disc parameters**	
Presence of DH, no. (%)	9 (1.3)
Disc area by HRT (mm^2^)	2.64 ± 9.52
Linear cup-to-disc ratio by HRT (mm^3^)	0.62 ± 0.15
**Measured ONH parameters**	
Disc tilt ratio	1.12 ± 0.12
Disc torsion degree	3.73 ± 8.15
Disc-foveal angle	7.31 ± 3.58
PPA area (pixel area)	9734.15 ± 12,932.56
Prelamina thickness (μm)	121.60 ± 139.50
Lamina cribrosa thickness (μm)	204.16 ± 53.13
Lamina cribrosa depth-BMO (μm)	452.19 ± 152.25
Lamina cribrosa depth-PPS (μm)	382.12 ± 112.54
Follow-up duration (m)	69.39 ± 7.77

DM, diabetes mellitus; HTN, systemic hypertension; OCT, optical coherence tomography; pRNFL, peripapillary retinal nerve fiber layer; mGC-IPL, macular ganglion cell-inner plexiform layer; IOP, intraocular pressure; VF, visual field; MD, mean deviation; PSD, pattern standard deviation; dB, decibel; MD, mean deviation; SAP, standard automated perimetry; DH, disc hemorrhage; HRT, Heidelberg retinal tomograph; ONH, optic nerve head; PPA, peripapillary atrophy; BMO, Bruch’s membrane opening; PPS, peripapillary sclera.

Data are mean ± standard deviation unless otherwise indicated.

**Table 2:** Comparison between NTG suspects that did and did not progress to NTG.

Variables	Progression to NTG	Non-progression to NTG	*P* value
	(n = 86)	(n = 598)	
**Demographics**			
Age at diagnosis (y)	55.93 ± 15.20	51.66 ± 16.02	0.019*
Female, no. (%)	49 (56.9)	395 (66.0)	0.096^†^
Family history of glaucoma, no. (%)	16 (18.6)	60 (10.0)	**0.027**^†^
Systemic demographics			
Medication of DM, no. (%)	10 (11.6)	32 (5.4)	0.051^†^
Medication of HTN, no. (%)	27 (31.4)	84 (14.0)	** < 0.001**^†^
**Ocular demographics**			
Best corrected visual acuity	0.91 ± 0.12	0.93 ± 0.12	0.092*
Axial length (mm)	24.92 ± 1.83	24.43 ± 2.02	0.031*
Central corneal thickness (μm)	546.21 ± 38.27	546.38 ± 45.71	0.974*
IOP parameters			
Baseline IOP (mmHg)	16.51 ± 3.64	15.92 ± 3.66	0.160*
Mean follow-up IOP (mmHg)	15.87 ± 3.05	16.73 ± 4.31	0.128*
**OCT parameters**			
Baseline average pRNFL thickness (μm)	87.05 ± 8.44	91.72 ± 9.54	< 0.001*
Baseline average mGC/IPL thickness (μm)	75.82 ± 8.35	78.90 ± 10.19	0.017*
VF parameters			
Baseline MD of SAP (dB)	− 1.60 ± 1.89	− 1.25 ± 1.83	0.093*
Baseline PSD of SAP (dB)	2.19 ± 1.23	1.78 ± 0.77	< 0.001*
**Disc parameters**			
Presence of DH, no. (%)	4 (4.7)	5 (0.8)	**0.018**^†^
Disc area by HRT (mm^2^)	2.32 ± 0.59	2.70 ± 10.26	0.735*
Linear cup-to-disc ratio by HRT (mm^2^)	0.63 ± 0.15	0.62 ± 0.15	0.444*
**Measured ONH parameters**			
Disc tilt ratio	1.22 ± 0.35	1.18 ± 0.18	0.252*
Disc torsion degree	− 1.96 ± 16.13	1.57 ± 12.11	0.016*
Disc-foveal angle	7.47 ± 4.64	28.29 ± 509.50	0.705*
PPA area (pixel area)	21,844.51 ± 29,260.82	15,533.89 ± 21,359.12	0.015*
Prelamina thickness (μm)	77.27 ± 42.26	94.70 ± 87.85	0.277*
Lamina cribrosa thickness (μm)	191.24 ± 45.97	215.24 ± 56.87	0.001*
Lamina cribrosa depth-BMO (μm)	464.93 ± 154.11	451.94 ± 139.95	0.658*
Lamina cribrosa depth-PPS (μm)	397.32 ± 107.58	382.46 ± 111.24	0.567*
Peripapillary choroidal thickness (μm)	142.34 ± 39.23	145.72 ± 42.25	0.720*
Follow-up duration (m)	71.83 ± 9.81	67.18 ± 6.13	0.540*

DM, diabetes mellitus; HTN, systemic hypertension; OCT, optical coherence tomography; pRNFL, peripapillary retinal nerve fiber layer; mGC-IPL, macular ganglion cell-inner plexiform layer; IOP, intraocular pressure; VF, visual field; MD, mean deviation; PSD, pattern standard deviation; dB, decibel; MD, mean deviation; SAP, standard automated perimetry; DH, disc hemorrhage; HRT, Heidelberg retinal tomograph; ONH, optic nerve head; PPA, peripapillary atrophy; BMO, Bruch’s membrane opening; PPS, peripapillary sclera.

Data are mean ± standard deviation unless otherwise indicated.

*Student’s *t *test.

^†^Chi-square test.

Data are mean ± standard deviation unless otherwise indicated.

Factors with statistical significance are shown in bold.

Cox regression analysis was performed to assess the baseline risk factors associated with conversion to NTG in NTG suspects (Table 3). Taking medication for diabetic mellitus (DM, *P* = 0.023) and systemic hypertension (*P* = 0.004), worse PSD of the VF (*P* < 0.001), thinner baseline average peripapillary RNFL (*P* < 0.001), greater inferotemporal direction of disc torsion (*P* = 0.001), and classification into the lower LC thickness group (*P* = 0.001) were significantly associated with NTG conversion in the univariate analysis. Among these factors, having family history of glaucoma (HR 1.380; 95% CI 1.002–1.723, *P* = 0.003), taking medication for systemic hypertension (HR 2.506; 95% CI 1.321–4.754; *P* = 0.005), thinner baseline average RNFL thickness (HR 0.966; 95% CI 0.941–0.991; *P* = 0.008), greater inferotemporal direction of disc torsion (HR 0.982; 95% CI 0.964–0.999; *P* = 0.042), and classification into the lower LC thickness group (HR 2.619; 95% CI 1.522–4.506; *P* = 0.001) were significant risk factors in multivariate analysis.

**Table 3:** Factors associated with progression to NTG in NTG suspect patients.

Variables	Univariate		Multivariate	
	HR (95% CI)	*P* value	HR (95% CI)	*P* value
Age (y)	1.009 (0.995–1.024)	0.227		
Female gender	0.745 (0.427–1.025)	0.207		
Family history of glaucoma	1.600 (0.933–2.744)	0.088	1.380 (1.002–1.723)	**0.003**
Medication of DM	2.343 (1.122–4.891)	0.023	1.235 (0.406–3.756)	0.71
Medication of HTN	1.991 (1.245–3.183)	0.004	2.506 (1.321–4.754)	**0.005**
History of migraine	2.001 (0.490–8.166)	0.334		
Best corrected visual acuity	0.453 (0.100–2.061)	0.306		
Axial length (mm)	1.069 (0.944–1.211)	0.291	1.053 (0.883–1.256)	0.566
Central corneal thickness (μm)	0.998 (0.993–1.003)	0.401		
Mean baseline IOP (mmHg)	1.012 (0.955–1.072)	0.692		
Mean follow-up IOP (mmHg)	1.030 (0.936–1.133)	0.541		
Baseline MD of SAP (for each dB worse)	0.911 (0.832–0.996)	0.042	0.932 (0.810–1.073)	0.327
Baseline PSD of SAP (for each dB worse)	1.348 (1.147–1.584)	< 0.001	1.240 (0.930–1.651)	0.142
Baseline average pRNFL thickness (μm)	0.959 (0.939–0.980)	< 0.001	0.966 (0.941–0.991)	**0.008**
Baseline average mGC/IPL thickness (μm)	0.983 (0.961–1.005)	0.136		
Presence of DH	1.923 (0.702–5.267)	0.203		
Disc area by HRT	0.989 (0.921–1.062)	0.763		
Linear cup-to-disc ratio by HRT	0.959 (0.195–4.728)	0.959		
Disc tilt	1.979 (0.771–5.083)	0.156		
Disc torsion	0.983 (0.974–0.993)	0.001	0.982 (0.964–0.999)	**0.042**
Disc-foveal angle	0.969 (0.916–1.025)	0.272		
PPA area	1.000 (1.000–1.000)	0.143		
Prelamina thickness	0.998 (0.992–1.004)	0.537		
Lower group of lamina thickness	2.246 (1.372–3.676)	0.001	2.619 (1.522–4.506)	**0.001**
Higher half group of lamina depth-PPS	1.472 (0.766–2.152)	0.62		

HR hazard ratio; CI, confidence interval; DM, diabetes mellitus; HTN, systemic hypertension; pRNFL, peripapillary retinal nerve fiber layer; mGC-IPL, macular ganglion cell-inner plexiform layer; IOP, intraocular pressure; MD, mean deviation; PSD, pattern standard deviation; dB, decibel; MD, mean deviation; SAP, standard automated perimetry; DH, disc hemorrhage; HRT, Heidelberg retinal tomograph; PPA, peripapillary atrophy; PPS, peripapillary sclera.

Factors with* P* < 0.1 in univariate analysis were included in multivariate analysis.

Data are mean ± standard deviation unless otherwise indicated.

Factors with statistical significance are shown in bold.

NTG suspects were divided into a non-myopic (n = 273) and myopic group (n = 411). In total, 34 (12.5%) of 273 non-myopic NTG suspects and 52 (12.7%) of 411 myopic NTG suspects converted to NTG. The NTG conversion rate was not significantly different between the non-myopic and myopic NTG suspects (*P* = 0.870). Non-myopic NTG suspects that converted to NTG were older (*P* = 0.002) and more frequently on medication for systemic hypertension (*P* < 0.001), and had a thinner baseline average peripapillary RNFL (*P* < 0.001), higher rate of disc hemorrhage during the follow-up period (*P* = 0.022), and thinner LC (*P* = 0.018) than non-myopic eyes that did not convert to NTG (Table 4). Myopic NTG suspects who converted to NTG had a higher frequency of family history of glaucoma (*P* = 0.008), longer axial length (*P* = 0.011), higher baseline IOP (*P* = 0.025), thinner baseline average peripapillary RNFL (*P* = 0.015) and macular GC/IPL thickness (*P* = 0.023), worse baseline PSD of the VF (*P* < 0.001), greater inferotemporal direction of disc torsion (*P* = 0.002), larger PPA area (*P* = 0.005), and thinner LC thickness (*P* < 0.001) than myopic eyes that did not convert to NTG (Table 5).

**Table 4:** Comparison between 273 NTG suspects without myopia that did and did not progress to NTG.

Variables	Non-myopic eyes with progression (n = 34)	Non-myopic eyes without progression (n = 239)	*P* value
**Demographics**			
Age at diagnosis (y)	66.62 ± 7.58	59.04 ± 14.09	0.002*
Female, no. (%)	29 (85.3)	199 (83.3)	0.961^†^
Family history of glaucoma, no. (%)	4 (11.8)	23 (9.6)	0.696^†^
**Systemic demographics**			
Medication of DM, no. (%)	5 (14.7)	20 (8.4)	0.513^†^
Medication of HTN, no. (%)	19 (55.9)	54 (22.6)	** < 0.001**^†^
**Ocular demographics**			
Best corrected visual acuity			
Axial length (mm)	23.19 ± 0.61	23.17 ± 0.56	0.903*
Central corneal thickness (μm)	543.35 ± 34.75	540.70 ± 49.37	0.763*
**IOP parameters**			
Baseline IOP (mmHg)	14.88 ± 3.79	15.44 ± 3.39	0.374*
Mean follow-up IOP (mmHg)	14.19 ± 2.94	15.95 ± 5.49	0.282*
**OCT parameters**			
Baseline average pRNFL thickness (μm)	86.37 ± 7.71	93.51 ± 9.18	< 0.001*
Baseline average mGC/IPL thickness (μm)	79.25 ± 8.92	81.30 ± 8.79	0.275*
**VF parameters**			
Baseline MD of SAP (dB)	− 1.05 ± 2.11	− 0.99 ± 1.60	0.860*
Baseline PSD of SAP (dB)	1.91 ± 0.98	1.76 ± 0.65	0.213*
**Disc parameters**			
Presence of DH, no. (%)	2 (5.9%)	2 (0.8%)	**0.022**^†^
Disc area by HRT (mm^2^)	2.25 ± 0.38	2.30 ± 0.42	0.496*
Linear cup-to-disc ratio by HRT (mm^2^)	0.63 ± 0.11	0.63 ± 0.13	0.893*
**Measured ONH parameters**			
Disc tilt ratio	1.08 ± 0.13	1.09 ± 0.11	0.550*
Disc torsion degree	1.81 ± 14.56	2.68 ± 13.77	0.731*
Disc-foveal angle	7.69 ± 4.56	7.41 ± 6.39	0.804*
PPA area (pixel area)	9,811.42 ± 9,744.33	9,431.57 ± 16,409.27	0.894*
Prelamina thickness	71.81 ± 39.87	72.41 ± 59.29	0.971*
Lamina cribrosa thickness (μm)	186.97 ± 38.97	196.50 ± 48.33	0.496*
Lamina cribrosa depth-BMO (μm)	418.66 ± 178.69	420.39 ± 133.76	0.970*
Lamina cribrosa depth-PPS (μm)	365.44 ± 102.32	373.72 ± 105.47	0.424*
Peripapillary choroidal thickness (μm)	153.18 ± 41.24	155.32 ± 37.96	0.652*
Follow-up duration (m)	73.44 ± 5.82	69.26 ± 8.39	0.716*

DM, diabetes mellitus; HTN, systemic hypertension; OCT, optical coherence tomography; pRNFL, peripapillary retinal nerve fiber layer; mGC-IPL, macular ganglion cell-inner plexiform layer; IOP, intraocular pressure; VF, visual field; MD, mean deviation; PSD, pattern standard deviation; dB, decibel; MD, mean deviation; SAP, standard automated perimetry; DH, disc hemorrhage; HRT, Heidelberg retinal tomograph; ONH, optic nerve head; PPA, peripapillary atrophy; BMO, Bruch’s membrane opening; PPS, peripapillary sclera.

Data are mean ± standard deviation unless otherwise indicated.

*Student’s *t *test.

^†^Chi-square test.

Data are mean ± standard deviation unless otherwise indicated.

Factors with statistical significance are shown in bold.

**Table 5:** Comparison between 411 NTG suspects with myopia that did and did not progress to NTG.

Variables	Myopic eyes with progression (n = 52)	Myopic eyes without progression (n = 359)	*P* value
**Demographics**			
Age at diagnosis (y)	48.60 ± 14.93	46.61 ± 15.35	0.383*
Female, no. (%)	19 (36.5)	193 (53.8)	0.125^†^
Family history of glaucoma, no. (%)	12 (23.1)	37 (10.3)	**0.008**^†^
**Systemic demographics**			
Medication of DM, no. (%)	4 (7.7)	12 (3.3)	0.130^†^
Medication of HTN, no. (%)	7 (13.5)	29 (8.1)	0.199^†^
**Ocular demographics**			
Best corrected visual acuity	0.92 ± 0.13	0.93 ± 0.13	0.199*
Axial length (mm)	26.05 ± 1.41	25.26 ± 2.19	**0.011**^†^
Central corneal thickness (μm)	550.34 ± 39.48	550.51 ± 42.88	0.979*
**IOP parameters**			
Baseline IOP (mmHg)	17.44 ± 3.95	15.16 ± 3.11	0.025*
Mean follow-up IOP (mmHg)	15.16 ± 3.11	17.03 ± 3.95	0.066*
**OCT parameters**			
Baseline average pRNFL thickness (μm)	87.06 ± 8.84	90.51 ± 9.60	0.015*
Baseline average mGC/IPL thickness (μm)	73.74 ± 7.32	77.56 ± 10.68	0.023*
**VF parameters**			
Baseline MD of SAP (dB)	− 1.98 ± 1.68	− 1.44 ± 1.94	0.053*
Baseline PSD of SAP (dB)	2.38 ± 1.37	1.80 ± 0.85	< 0.001*
**Disc parameters**			
Presence of DH, no. (%)	2 (3.8)	3 (0.8)	0.064^†^
Disc area by HRT (mm^2^)	2.37 ± 0.69	2.96 ± 13.23	0.748*
Linear cup-to-disc ratio by HRT (mm^2^)	0.64 ± 0.17	0.61 ± 0.16	0.311*
**Measured ONH parameters**			
Disc tilt ratio	1.32 ± 0.42	1.23 ± 0.21	0.160*
Disc torsion degree	− 4.56 ± 16.77	0.83 ± 10.83	0.002*
Disc-foveal angle	7.33 ± 4.74	42.19 ± 657.56	0.705*
PPA area (pixel area)	30,102.51 ± 34,929.21	19,607.78 ± 23,249.71	0.005*
Prelamina thickness	82.41 ± 44.98	107.60 ± 98.96	0.313*
Lamina cribrosa thickness (μm)	190.25 ± 45.57	224.66 ± 59.05	< 0.001*
Lamina cribrosa depth-BMO (μm)	508.48 ± 115.77	470.21 ± 141.34	0.312*
Lamina cribrosa depth-PPS (μm)	462.34 ± 102.32	433.72 ± 101.96	0.446*
Peripapillary choroidal thickness (μm)	139.16 ± 42.55	141.35 ± 40.74	0.527*
Follow-up duration (m)	72.77 ± 8.28	68.91 ± 7.06	0.823*

DM, diabetes mellitus; HTN, systemic hypertension; OCT, optical coherence tomography; pRNFL, peripapillary retinal nerve fiber layer; mGC-IPL, macular ganglion cell-inner plexiform layer; IOP, intraocular pressure; VF, visual field; MD, mean deviation; PSD, pattern standard deviation; dB, decibel; MD, mean deviation; SAP, standard automated perimetry; DH, disc hemorrhage; HRT, Heidelberg retinal tomograph; ONH, optic nerve head; PPA, peripapillary atrophy; BMO, Bruch’s membrane opening; PPS, peripapillary sclera.

Data are mean ± standard deviation unless otherwise indicated.

*Student’s* t *test.

^†^Chi-square test.

Data are mean ± standard deviation unless otherwise indicated.

Factors with statistical significance are shown in bold.

To assess the differences in baseline factors associated with NTG conversion in NTG suspects, we performed Cox regression analysis in the myopic and non-myopic groups. Taking medication for systemic hypertension (HR 2.933; 95% CI 1.199–7.254; *P* = 0.022), thinner baseline average peripapillary RNFL (HR 0.943; 95% CI 0.904–0.985; *P* = 0.008), and classification into the lower LC thickness group (HR 4.775; 95% CI 1.345–7.626; *P* = 0.045) were significant baseline risk factors for NTG conversion in non-myopic NTG suspects in multivariate analysis (Table 6). Family history of glaucoma (HR 3.041; 95% CI 1.477–6.262; *P* = 0.003), worse baseline SAP of the VF (HR 1.345; 95% CI 1.014–1.784; *P* = 0.040), greater inferotemporal direction of disc torsion (HR 0.982; 95% CI 0.975–0.992; *P* = 0.024), and classification into the lower LC thickness group (HR 5.344; 95% CI 2.725–10.480; *P* < 0.001) were significant baseline risk factors for NTG conversion in myopic NTG suspects in multivariate analysis (Table 7).

**Table 6:** Factors associated with progression to NTG in NTG suspect patients without myopia.

Variables	Univariate		Multivariate	
	HR (95% CI)	*P* value	HR (95% CI)	*P* value
Age (y)	1.045 (1.009–1.083)	**0.014**	1.024 (0.978–1.065)	0.422
Female gender	1.478 (0.599–3.645)	0.396		
Family history of glaucoma	1.235 (0.473–3.221)	0.666		
Medication of DM	2.478 (0.842–7.292)	0.099	2.390 (0.782–4.830)	0.254
Medication of HTN	2.581 (1.323–5.033)	**0.005**	2.933 (1.199–7.254)	**0.022**
History of migraine	22.096 (0.026–50.832)	0.367		
Best corrected visual acuity	0.986 (0.084–11.632)	0.986		
Axial length (mm)	0.774 (0.424–1.412)	0.403		
Central corneal thickness (μm)	0.998 (0.991–1.004)	0.482		
Mean baseline IOP (mmHg)	0.967 (0.878–1.064)	0.492		
Mean follow-up IOP (mmHg)	0.986 (0.843–1.154)	0.864		
Baseline MD of SAP (for each dB worse)	0.928 (0.758–1.137)	0.471		
Baseline PSD of SAP (for each dB worse)	1.422 (0.930–2.174)	0.104		
Baseline average pRNFL thickness (μm)	0.944 (0.914–0.976)	**0.001**	0.943 (0.904–0.985)	**0.008**
Baseline average mGC/IPL thickness (μm)	0.974 (0.934–1.016)	0.218		
Presence of DH	1.823 (0.662–5.018)	0.571		
Disc area	0.852 (0.388–2.187)	0.852		
Linear cup-to-disc ratio	0.266 (0.017–4.036)	0.34		
Disc tilt	0.479 (0.016–14.613)	0.673		
Disc torsion	0.986 (0.970–1.002)	**0.093**	0.975 (0.942–1.015)	0.326
Disc-foveal angle	0.996 (0.923–1.075)	0.923		
PPA area	1.000 (1.000–1.000)	0.382		
Prelamina thickness	0.999 (0.990–1.008)	0.863		
Lower group of lamina thickness	3.856 (1.670–7.234)	0.005	4.775 (1.345–7.626)	**0.045**
Higher half group of lamina depth-PPS	1.024 (0.462–1.403)	0.792		

HR, hazard ratio; CI, confidence interval; DM, diabetes mellitus; HTN, systemic hypertension; pRNFL, peripapillary retinal nerve fiber layer; mGC-IPL, macular ganglion cell-inner plexiform layer; IOP, intraocular pressure; MD, mean deviation; PSD, pattern standard deviation; dB, decibel; MD, mean deviation; SAP, standard automated perimetry; DH, disc hemorrhage; PPA, peripapillary atrophy; PPS, peripapillary sclera.

Factors with* P* < 0.1 in univariate analysis were included in multivariate analysis.

Data are mean ± standard deviation unless otherwise indicated.

Factors with statistical significance are shown in bold.

**Table 7:** Factors associated with progression to NTG in NTG suspect patients with myopia.

Variables	Univariate		Multivariate	
	HR (95% CI)	*P* value	HR (95% CI)	*P* value
Age (y)	0.998 (0.980–1.017)	0.864		
Female gender	0.622 (0.352–1.099)	0.102		
Family history of glaucoma	2.061 (1.062–4.000)	**0.032**	3.041 (1.477–6.262)	**0.003**
Medication of DM	2.219 (0.783–6.287)	0.134		
Medication of HTN	1.602 (0.716–3.585)	0.252		
History of migraine	1.008 (0.239–4.254)	0.991		
Best corrected visual acuity	0.181 (0.025–1.331)	0.093	0.227 (0.031–1.649)	0.143
Axial length (mm)	1.188 (0.994–1.420)	0.059	1.033 (0.801–1.333)	0.8
Central corneal thickness (μm)	0.998 (0.991–1.005)	0.542		
Mean baseline IOP (mmHg)	1.046 (0.968–1.129)	0.254		
Mean follow-up IOP (mmHg)	1.074 (0.950–1.214)	0.253		
Baseline MD of SAP (for each dB worse)	0.899 (0.812–0.996)	**0.042**	0.985 (0.842–1.152)	0.846
Baseline PSD of SAP (for each dB worse)	1.374 (1.146–1.647)	**0.001**	1.345 (1.014–1.784)	**0.04**
Baseline average pRNFL thickness (μm)	0.968 (0.941–0.996)	**0.025**	0.979 (0.950–1.009)	0.176
Baseline average mGC/IPL thickness (μm)	0.981 (0.955–1.006)	0.139		
Presence of DH	3.039 (0.728–12.698)	0.128		
Disc area	0.989 (0.918–1.065)	0.773		
Linear cup-to-disc ratio	1.625 (0.218–12.110)	0.636		
Disc tilt	2.762 (0.975–7.826)	0.056	0.825 (0.168–4.046)	0.813
Disc torsion	0.973 (0.956–0.991)	**0.003**	0.982 (0.975–0.992)	**0.024**
Disc-foveal angle	0.953 (0.889–1.022)	0.179		
PPA area	1.000 (1.000–1.000)	0.148		
Prelamina thickness	0.998 (0.990–1.006)	0.623		
Lower group of lamina thickness	4.224 (2.343–7.618)	** < 0.001**	5.344 (2.725–10.480)	** < 0.001**
Higher half group of lamina depth-PPS	1.320 (0.524–2.216)	0.53		

HR, hazard ratio; CI, confidence interval; DM, diabetes mellitus; HTN, systemic hypertension; pRNFL, peripapillary retinal nerve fiber layer; mGC-IPL, macular ganglion cell-inner plexiform layer; IOP, intraocular pressure; MD, mean deviation; PSD, pattern standard deviation; dB, decibel; MD, mean deviation; SAP, standard automated perimetry; DH, disc hemorrhage; PPA, peripapillary atrophy; PPS, peripapillary sclera.

Factors with* P* < 0.1 in univariate analysis were included in multivariate analysis.

Data are mean ± standard deviation unless otherwise indicated.

Factors with statistical significance are shown in bold.

**Figure 1:**
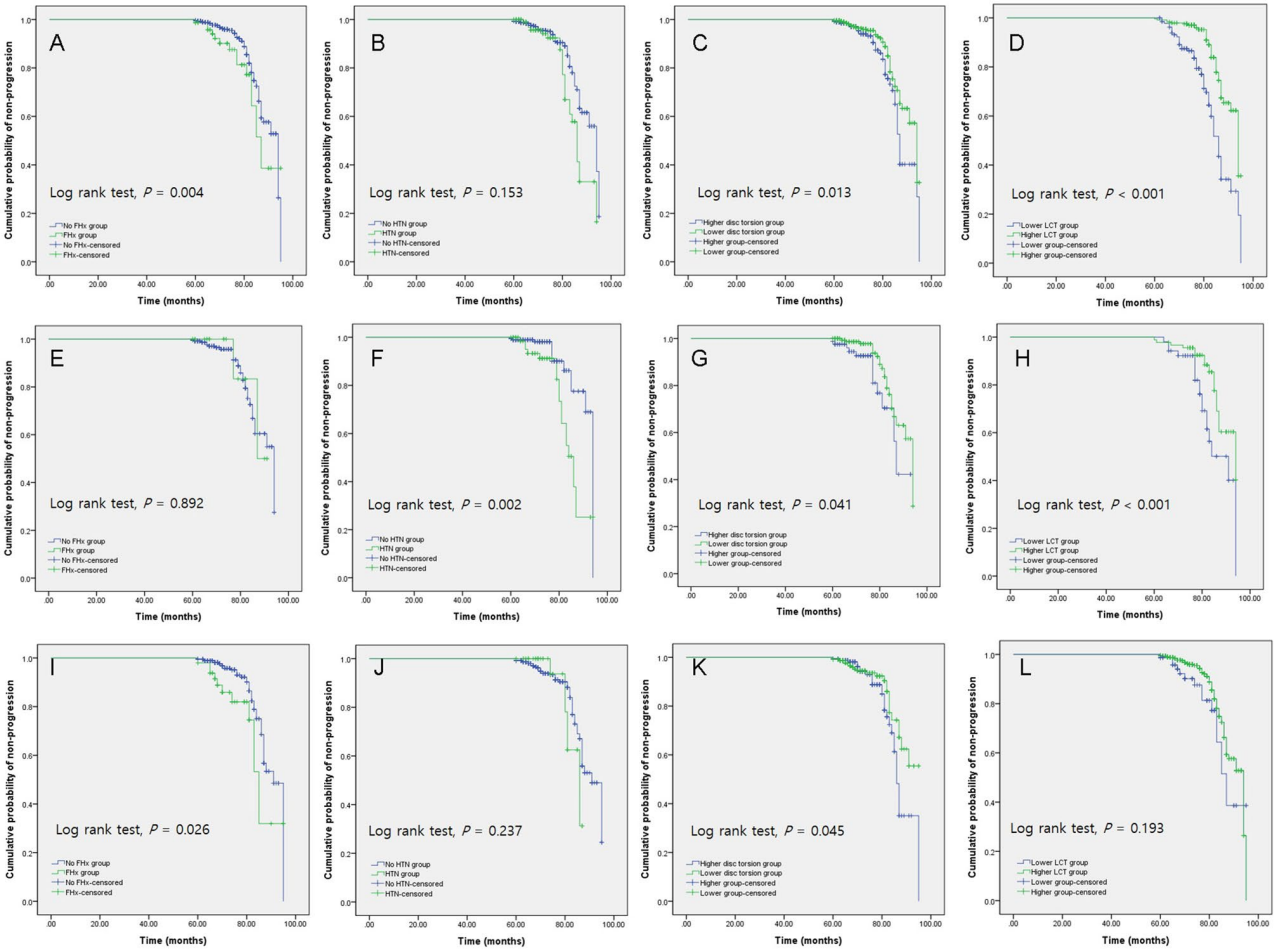
Kaplan–Meier survival analysis of normal-tension glaucoma (NTG) conversion in NTG suspects according to family history of glaucoma (**A**, **E**, **I**); medication for systemic hypertension (HTN) (**B**, **F**, **J**); direction of disc torsion (**C**, **G**, **K**); lamina cribrosa thickness measured on spectral-domain optical coherence tomography scans, classified according to a cut-off value of 180.5 μm (calculated based on the data of the present cohort) (**D**, **H**, **L**). (**A**–**D**), all NTG suspects; (**E**–**H**), NTG suspects without myopia; (**I**–**L**), NTG suspects with myopia.

The results of Kaplan–Meier survival analyses for NTG conversion in NTG suspects are shown in Fig. 1. Overall, the NTG suspects with a family history of glaucoma (*P* = 0.004; log rank test; Fig. 1A), disc torsion to the inferotemporal direction (*P* = 0.013; log rank test; Fig. 1C), and baseline LC thickness < 180.5 μm (*P* < 0.001; log rank test; Fig. 1D) showed significant differences in the cumulative probability of survival. In the NTG suspect group without myopia, NTG suspects on medication for systemic hypertension (*P* = 0.002; log rank test; Fig. 1F) and with baseline LC thickness < 180.5 μm (*P* = 0.038; log rank test; Fig. 1H) showed significant differences in the cumulative probability of survival. In the NTG suspect group with myopia, NTG suspects with family history of glaucoma (*P* = 0.026; log rank test; Fig. 1I) and disc torsion in the inferotemporal direction (*P* = 0.045; log rank test; Fig. 1K) showed significant differences in cumulative probability of survival.

## Discussion

The rate of progression and conversion to NTG in NTG suspects was 12.6% after a mean follow-up period of about 5.78 years in the present study. The Advanced Imaging for Glaucoma Study showed that 8.5% of OHT eyes with normal-appearing discs progressed to glaucoma, whereas 31.8% of OHT eyes with ONH/RNFL defects, and 42.9% with ONH/RNFL defects (and with IOP in the normal range) progressed to glaucoma during similar follow-up period with the present study^7^. In other studies, normotensive preperimetric glaucoma progressed to glaucomatous VF damage in 13% to 57.7% of cases after a 3–to 5-year follow-up^4–8^. This study was a multicenter study conducted prospectively including large number of NTG suspects than other previous studies that were mostly retrospective designs with limited number of suspects. To identify NTG suspects with IOP within the normal range and a normal VF, the inclusion criteria in this study were eyes with a larger concentric cup-to-disc ratio and/or neuroretinal rim thinning, but without focal rim notching or localized RNFL defects. Thus, we could identify related risk factor for developing NTG in suspected individuals, less likely in the stage of preperimetric glaucoma. In addition to the VF criteria, we used the presence of localized RNFL defects to determine the likelihood of conversion to glaucoma based on red-free fundus photographs. This may detect NTG conversion before the presence of VF damage since VF is known to detect glaucoma development later on the disease course. The baseline risk factors identified in the present study may identify pure NTG suspects with higher risk and less likely to include suspects in the preperimetric NTG stage. In addition, we included suspects without suspicious RNFL defect and had normal RNFL on baseline OCT. Therefore, suspects included in the present study represents disc suspects, which means suspects with only suspicious optic disc appearances that we encounter frequently in clinic. The rate of conversion was somewhat lower than in studies evaluating VF conversion in normotensive preperimetric glaucoma, but was comparable or slightly higher than that of the OHT cases with normal-appearing discs, indicating that, even with IOP within the normal range, suspicious optic disc appearance contributes to the development of glaucoma similar to an elevated IOP.

Identifying predictive risk factors for the development of NTG in NTG suspects are clinically important. Until now, we could only adopt findings from OHTS to classify glaucoma risks in NTG suspects. However, this is not precise since OHTS only included suspects with high IOP without any findings in the optic disc. IOP factors were important in developing glaucoma to suspects with high IOP which was found in the OHTS. No IOP factors, including baseline IOP and mean follow-up IOP, were significant risk factors for NTG conversion in NTG suspects. Instead, medication for systemic hypertension, axial length, baseline MD and PSD of the VF, baseline average peripapillary RNFL thickness, disc torsion, and thinner LC at baseline were significant predictors. The risk factors can be summarized as baseline structural ocular characteristics (baseline axial length, disc torsion, LC thickness), systemic hypertension, and baseline VF and RNFL parameters. Baseline structural ocular characteristics related to eyeball elongation (disc torsion) were more important in myopic NTG suspects, whereas baseline structural characteristics of the ONH (LC thickness) and systemic hypertension were more important in NTG suspects without myopia.

**Figure 2:**
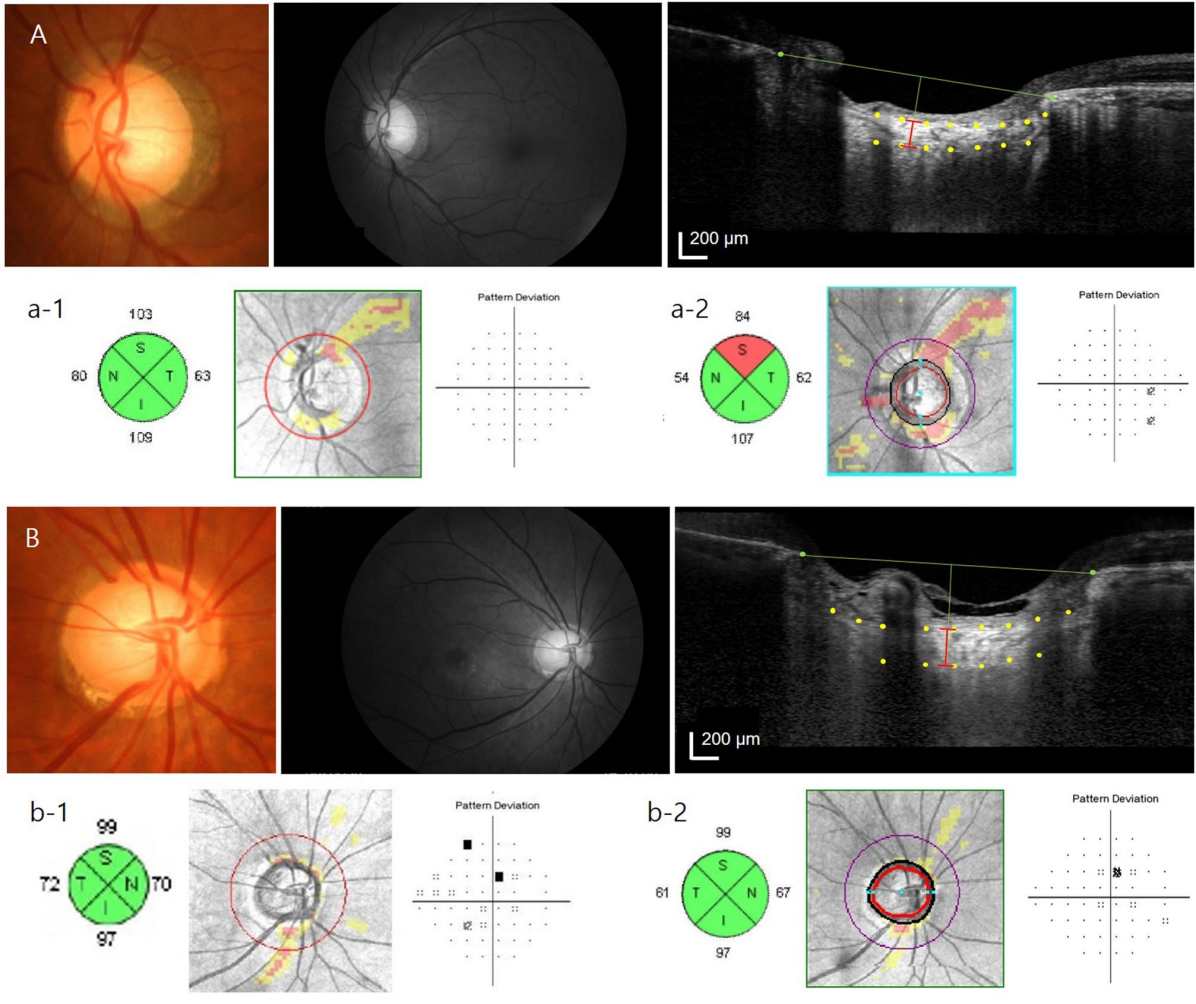
Representative cases showing the degree of disc torsion contributes to normal-tension glaucoma (NTG) conversion in NTG suspects with myopia. (**A**)**,** A 32-year-old female with large cup-to-disc ratio in the left eye who had been follow-up for 9 years. Fundus photography shows disc torsion to the inferotemporal region for about -7.42° (**a-1**). Optic nerve head scan shows laminar cribrosa thickness of 221 μm (**a-2**). Progression to NTG was detected by the presence of localized retinal nerve fiber layer defect at the inferotemporal region (**a-4**) compared to baseline (a-3). (**B)**, A 42-year-old male with large cup-to-disc ratio in the left eye who had been follow-up for 9 years. Fundus photography shows no disc torsion (**b-1**). Optic nerve head scan shows laminar cribrosa thickness of 216 μm (**b-2**). Progression to NTG was not detected during the follow-up period (**b-3** and **b-4**).

The LC is thought to be important in the pathogenesis of glaucoma. Various features of the LC were reported to change during the process of glaucoma development, and may be related to the development and progression of the disease^11–13^. Our group proposed the importance of the LC in NTG, especially its thickness, and reported that the LC was thinner in NTG patients compared to patients in a similar stage of primary open-angle glaucoma^14–19^. The LC was thinner in eyes with unilateral VF defects, when comparing both eyes of NTG patients^20^. There are still issues associated with imaging of the LC, and determining its thickness based on SD-OCT images. However, it is an important feature assessed in many studies^21–30^. A thinner baseline LC was reported to contribute to future glaucoma progression^16,27,28^. In addition, a thinner baseline LC may be important in the development of glaucoma. The baseline LC thickness was shown to be thinner in certain ethnic groups, which may contribute to a higher likelihood of glaucoma^24^. A thinner baseline LC was significantly associated with NTG conversion in NTG suspects in the present study. As NTG in prevalent in Korea and Japan, and where most cases are related to myopia, we hypothesized that some components of the extracellular matrix in the LC may contribute to the pathogenesis of NTG^16^. Although further studies are needed, we found that evaluating the LC at baseline in our cohort of NTG suspects was important for predicting NTG conversion. This finding should be important to consider in managing cases with a suspicious optic disc. Representative cases are shown in Fig. 2. Two NTG suspects show enlarged cup-to-disc ratio and neuroretinal rim thinning with normal-range IOP during follow-up. Both cases have baseline RNFL thickness in the normal-range on the quadrant map of RNFL OCT without definite focal RNFL defect on red-free photographs. However, former NTG suspect with thinner LC on ONH scan shows progression to preperimetric NTG (Fig. 2A), which is not observed in the latter case with thicker LC (Fig. 2B). Baseline LC thickness < 180.5 μm was the cut-off value for predicting NTG conversion in NTG suspects. Previously, we derived a cut-off value of 221 μm for discriminating between NTG and normal eyes in terms of LC thickness. These values were obtained from a different cohort and the present study included a larger number of myopic eyes, which could explain the thinner LCs. Additional features and measurements of the LC, other than its thickness and depth, are also emerging and being associated with glaucoma development^31,32^. Further studies considering various features of the LC parameters in glaucoma suspects are needed.

**Figure 3:**
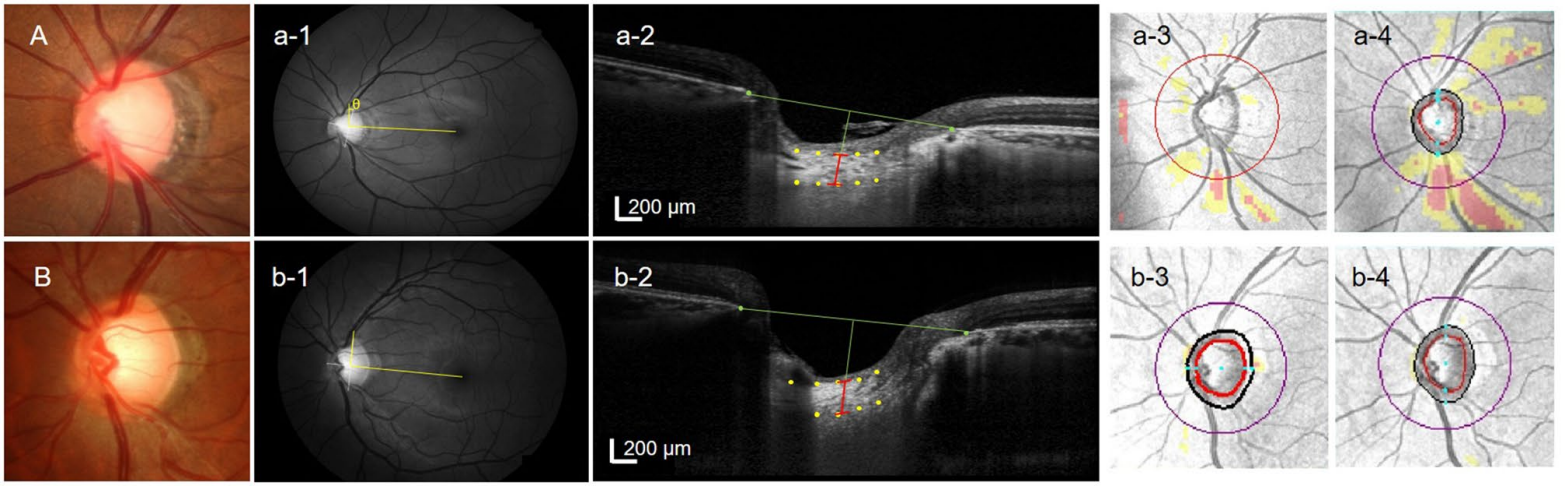
Representative cases showing the thinner lamina cribrosa (LC) contributes to normal-tension glaucoma (NTG) conversion in NTG suspects. (**A**)**,** A 62-year-old male with large cup-to-disc ratio in the left eye who had been follow-up for 9 years. Optic nerve head scan shows laminar cribrosa thickness of 127 μm. Progression to NTG was detected by the presence of localized retinal nerve fiber layer defect at the superotemporal region (**a-2**) compared to baseline (**a-1**). (**B**)**,** A 63-year-old male with large cup-to-disc ratio and neuroretinal rim thinning in the right eye who had been follow-up for 10 years. Optic nerve head scan shows laminar cribrosa thickness of 265 μm. Progression to NTG was not detected during the 10-year follow-up period (**b-1** and **b-1**).

Disc torsion in the inferotemporal direction, and the degree of disc torsion, were associated with NTG development in NTG suspects in this study, especially in eyes with myopia. The direction of disc torsion was reported to be related to the location of glaucomatous damage in myopic glaucoma eyes^10,33,34^. Eyeball elongation during the myopic process seems to influence the susceptibility of the retinal ganglion cell axons, and the most deformed regions may show greater vulnerability to glaucomatous damage. This can also explain the link between glaucoma and myopia. However, there have been no studies in which myopic patients were followed up to determine the actual susceptibility and likelihood of glaucoma development, especially in terms of NTG. In the present study, the rate of NTG conversion was similar between myopic eyes with suspicious disc appearance and non-myopic suspects, but the risk factors were quite different between the two groups. Axial length and the degree of disc torsion are somewhat related, but do not always correlate^16^. Our previous studies suggested that for eyes of similar axial length, a greater degree of disc torsion and scleral deformation seemed to be more important in glaucoma^35,36^. In the present study, axial length was significantly associated with the development of NTG in myopic NTG suspects in univariate regression analysis, but not in multivariate analysis. Only the degree of disc torsion was significantly associated with NTG development in both univariate and multivariate regression analysis in myopic NTG suspects. This suggested that the degree of deformation of the posterior pole is more important than the eyeball elongation itself for determining the likelihood of NTG development in NTG suspects with myopia. In addition, disc torsion also showed a significant association with NTG conversion in non-myopic NTG suspects in univariate analysis. This factor was not significant in multivariate analysis, but the degree of eyeball deformation, in addition to axial length or myopia, could be important in the development of glaucoma. Representative cases are shown in Fig. 3. Two NTG suspects have similar LC thickness on baseline ONH OCT scans, but the former NTG suspect with larger degree of disc torsion to the inferotemporal direction shows progression to NTG (Fig. 3A), which the later NTG suspect without disc torsion did not progress to NTG (Fig. 3B).

Medication for systemic hypertension was a significant risk factor for NTG conversion in the present study. Medication for systemic hypertension would be expected to indicate a diagnosis thereof, but we nevertheless checked the medication prescriptions of patients for accurate diagnosis of systemic diseases. There have been many reports regarding the association between glaucoma and systemic hypertension. High and low diastolic blood pressure, and fluctuation thereof, were all shown to be related to the development and progression of glaucoma^37,38^. Nocturnal or orthostatic hypotension related to medication use in glaucoma patients with systemic hypertension was in turn thought to be related to glaucoma progression, causing a drop in ocular perfusion pressure that leads to ischemia–reperfusion injury of the ONH^39–41^. In this study, medication for systemic hypertension was especially important in NTG suspects without myopia who converted to NTG. This suggested that ischemia caused by systemic hypertension or medication in NTG suspects may contribute to the development of glaucoma in eyes with less profound structural weakness of the ONH or the posterior pole.

The present study had several limitations that must be taken into consideration when interpreting the results. First, there may have been issues defining the NTG suspects. In particular, the exclusion of eyes with a suspicious-appearing focal area of the disc rim, or suspicious focal RNFL defects, could have influenced the results. However, our intention was to identify risk factors for NTG suspects with IOP within the normal range, and therefore we applied highly specific and strict criteria for NTG conversion. This may have reduced the NTG conversion rate in the present study. There were also limitations in terms of the imaging modalities and measurement of LC thickness. The posterior border of the LC is not distinct in some eyes. As our cohort included a high percentage of myopic eyes with a thin LC, identifying the posterior border was less important. However, it is somewhat difficult to image the LC in myopic eyes because of the small cup area and disc tilting, which results in posterior shadowing and blocks the signals arising from the LC. Disc tilting could also influence the image quality and measurement reproducibility due to the scans obtained skewed relative to the perpendicular lines to the reference lines connecting the openings of the Bruch’s membrane. All of the myopic eyes included in our study had an enlarged cup-to-disc ratio, which reduced the difficulty of imaging the LC. We had to exclude images with obscure LC border at the bottom of the LC region. The measurement of LC depth could be influenced by the choroidal thickness which could also be variable between individuals and between myopic eyes. We added both LC depth from the reference planes of BMO and PPS trying the overcome this problem.

In conclusion, 12.6% of our NTG suspects converted to NTG during the 5–6-year follow-up period. NTG suspects taking medication for systemic hypertension, with disc torsion of the optic disc in the inferior direction, and thinner LC of the ONH at baseline were at greater risk of NTG conversion. Baseline risk factors, such as medication for systemic hypertension, disc torsion on retina/disc photographs, and thinner LC on ONH OCT scans, should be taken into consideration in the treatment planning and follow-up of NTG suspects.

## Methods

### Subjects

This study was a component of the Catholic Medical Center Glaucoma Suspect Cohort Study (CMC-GSCS), which commenced in 2009 at multiple centers affiliated with the Catholic Medical Center, South Korea. The work was approved by each participating hospital’s institutional review board (Institutional review board of the Seoul St. Mary’s Hospital, Yeouido St. Mary’s Hospital, St. Vincent’s Hospital, and Eunpyeong St. Mary’s Hospital) and was performed in accordance with all relevant tenets of the Declaration of Helsinki. We enrolled all consecutive eligible patients who were willing to participate, and all provided written informed consent.

All NTG suspect subjects enrolled in the CMC-GSCS underwent a complete ophthalmic examination, including detailed history-taking, detailed review of medical/ocular records and prescriptions, measurement of best-corrected visual acuity, refraction assessment, slit-lamp biomicroscopy, gonioscopy, Goldmann applanation tonometry, measurement of central corneal thickness by ultrasound pachymetry (Tomey Corp., Nagoya, Japan), measurement of axial length with ocular biometry (IOL Master; Carl Zeiss Meditec, Dublin, CA, USA), dilated stereoscopic examination of the optic disc, red-free fundus photography (Canon, Tokyo, Japan), VF examination using the Swedish interactive threshold algorithm Standard 24–2 (Humphrey; Carl Zeiss Meditec) and frequency-doubling technology (FDT) perimetry (Humphrey Matrix; Carl Zeiss Meditec), RNFL OCT (Cirrus; Carl Zeiss Meditec), and an enhanced depth imaging (EDI)-OCT scan of the ONH (Spectralis; Heidelberg Engineering, Heidelberg, Germany). All patients were followed up every 1–3 months with IOP measurement and optic disc evaluation. Disc photography, VF, and OCT examinations were performed every year. All disc hemorrhages (DH) occurring during follow-up were recorded. IOP was recorded at each visit. The mean IOP during the entire follow-up period was calculated by averaging all measurements.

One of the aims of the study was to predict conversion of NTG suspects to NTG. Therefore, we defined strict criteria for NTG suspects to minimize the inclusion of cases of preperimetric glaucoma at baseline. Eyes categorized as NTG suspects did not have an abnormal VF on pattern deviation plots and the glaucoma hemifield test (GHT), and either the suspected eye or the contralateral eye had ocular hypertension. Suspicious optic disc findings, such as neuroretinal rim thinning, excavation, or enlarged cup-to-disc ratio (≥ 0.6), had to be present on disc photograph. However, eyes with focal rim notching, localized RNFL defects on disc and red-free photographs, or color code abnormalities of quadrant map on RNFL OCT were not included.

Additional inclusion criteria were as follows: no history of glaucoma treatment, including medication, laser, or surgery; best-corrected visual acuity ≥ 20/40, spherical refraction within ± 6.0 diopters (D), cylinder correction within ± 3.0 D, axial length ≥ 30 mm, at least two reliable VF measurements (false-negatives < 15%, false-positives < 15%, and fixation losses < 20%), and mean deviation (MD) better than − 6.00 decibels (dB). The exclusion criteria were as follows: history of any retinal disease, including diabetic or hypertensive retinopathy; history of eye trauma or surgery with the exception of uncomplicated cataract surgery; history of refractive surgery; any optic nerve disease other than glaucoma; and history of systemic or neurological diseases that may affect the VF.

### Analysis of disc and fundus images for posterior pole profiles

To derive the posterior pole profiles, we measured disc tilt according to the disc ovality ratio, degree of disc torsion, peripapillary atrophy (PPA) area, and disc − foveal angle using disc and red-free RNFL photographs (Fig. 1). The measurement parameters were all described previously^10,42–46^.

**Figure 4:**
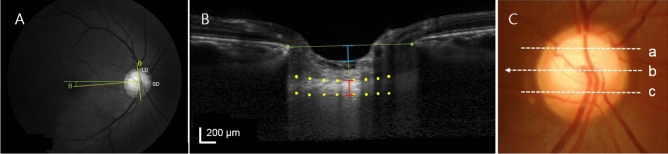
The optic disc morphological features (disc tilt, disc torsion, and disc-foveal angle) and optic nerve head (ONH) parameters (prelaminar thickness, lamina cribrosa [LC] depth, and LC thickness) were measured using disc and retinal nerve fiber layer photographs and B-scans from spectral-domain optical coherence tomography (SD-OCT). (**A**)**,** The disc tilt was determined using the disc ovality ratio. Disc ovality ratio was the ratio between the longest disc diameter (LD) and shortest disc diameter (SD) (**white dotted lines**). Disc torsion refers to the deviation of the long disc (LD) axis from the vertical meridian (*i.e*., the vertical line perpendicular to a reference line connecting the fovea and the disc center; yellow lines). The angle (**yellow θ**) between the vertical meridian and the long axis of the disc is the degree of torsion. The disc-foveal angle (**green θ**) is the angle between the optic disc and fovea as measured by the angle between the reference line (**yellow line**) and a horizontal line through the disc center (**green line**). (B**),** To measure the ONH parameters, the end of Bruch’s membrane was marked (green glyph) and connected by the reference line (green line). From the center of the reference line, a perpendicular line was drawn and measurements were made along this line. Prelaminar thickness was the distance between the center of the reference line to the anterior surface of the ONH (marked as blue section). LC thickness was the distance between the anterior and posterior borders of the LC which appears as a hyperreflective region at the bottom of the ONH (marked as red section). The LC depth was determined by measuring the distance from the center of the reference line to the anterior surface of the LC. ONH parameters were measured at three locations from each B-scans that scanned three regions (**a**, **b**, **c**) of the ONH and average of three values were used.

Disc and red-free RNFL photographs were obtained using non-mydriatic retinal camera operating under the standard settings (Kowa, Tokyo, Japan). The disc photographs and red-free images were evaluated independently in a random order, and in a masked fashion, by two of the authors (D.Y.S. and S.J.J.). The disc ovality ratio, degree of disc torsion, PPA area, and disc − foveal angle were measured on photographs using ImageJ software (version 1.40; NIH, Bethesda, MD, USA). The disc ovality index was determined according to the tilt ratio, defined as the ratio between the longest and shortest disc diameters (Fig. 4A, white dotted lines refer the longest and shortest disc diameters; LD = longest disc diameters; SD = shortest disc diameters)^47–49^. Disc torsion refers to the deviation of the long disc axis from the vertical meridian (*i.e*., the vertical line perpendicular to a reference line connecting the fovea and the disc center). The angle between the vertical meridian and the long axis of the disc is the degree of torsion (Fig. 4A, yellow lines refer to the reference line and the vertical line; yellow θ = degree of disc torsion)^50,51^. Positive and negative angles indicated the presence of superonasal and inferotemporal torsion, respectively. Beta zone PPA, defined as an inner crescent of chorioretinal atrophy with visible sclera and choroidal vessels, was plotted using a mouse-driven cursor to trace the disc and PPA margin directly onto the image. The pixel areas were calculated to define the PPA area. The disc − foveal angle was defined as the angle between the optic disc and fovea, i.e., the angle between the reference line and a horizontal line through the disc center, as described previously (Fig. 4A, green line refers to the horizontal line; green θ = disc − foveal angle)^46,52^. A negative value indicated that the fovea was located inferior to the optic disc, while a positive value indicated that it was superior to the optic disc.

### Analysis of optical coherence tomography images for determining optic nerve head parameters

The Heidelberg Spectralis OCT system provides up to 40,000 A-scans/s with a depth resolution of 7 μm in tissues, and a transverse resolution of 14 μm in images of ocular microstructures. EDI-OCT B-scans around the ONH (6-mm optic cube scans) were obtained using the Spectralis OCT system. Each section was obtained using eye tracking and incorporated an average of at least 35 OCT frames. Images with a quality score > 15 were obtained (~ 65–70 sections per eye). We measured the prelamina, lamina cribrosa (LC) thickness, and LC depth based on the averaged images. Measurements were made using the caliper function of the OCT software by two observers (D.Y.S. and S.J.J) in a blinded manner. Details of the ONH parameter measurement technique have been presented elsewhere^17,18,53^. Myopic eyes with tilting causing the images not clear at the bottom of the LC area were excluded.

Prelamina thickness was defined as the distance between the anterior border of the bottom of the cup to the anterior border of the hyperreflective region at the bottom of the ONH (Fig. 4B, blue lines refer the measurement of prelamina thickness). LC thickness was defined as the distance between the anterior and posterior borders of the hyperreflective region at the bottom of the ONH. Measurements were performed along a line perpendicular to the reference line, connecting the end of Bruch’s membrane to the center of the reference line (Fig. 4B, green glyph are at the ends of Bruch’s membrane and the green lines refer the reference line, red lines refer the measurement of LC thickness). These measurements were performed in the superior mid-peripheral, center, and inferior mid-peripheral regions, which were scanned throughout the ONH (Fig. 4C). The average of three measurements in each location was used to derive the LC thickness. The LC depth was determined by measuring the distance from the opening plane of Bruch’s membrane to the level of the anterior LC surface. The average of the three values was used as the LC depth. To avoid the influence of the choroidal thickness, peripapillary sclera (PPS) was used for the reference plane to measure the LC depth with the same images^54^. The PPS was defined by high signal intensity just beneath the choroid. The line joining the outermost points of the PPS on the B-scan image was defined as the PPS reference plane. The distance from the PPS reference plane to the level of the anterior LC surface was the LC depth defined by the PPS.

Peripapillary choroidal thickness were measured from the B-scans of the ONH. Inner scleral wall and posterior border of the retinal pigment epithelium were delineated to define the outer and inner boundaries of the choroid. The vertical distance between the two delineated lines were considered as peripapillary choroidal thickness.

### Corrected parameters by ocular magnification

Effect of ocular magnification was considered and posterior pole profiles and ONH parameters were corrected^55^. The relationship between the measured disc/RNFL photographs and OCT images diameter, Dm, and the true diameter on the fundus, Dt, can be expressed as: Dt = p × q × Dm; where p x q is the overall image magnification factor; p is that of the imaging system and q is that of the eye. The factor q can be determined: q = 0.01306 × (axial length–1.82). The factor p, omitting any effect arising from image distortion, can be readily calculated from the Bennet formula if the axial length at which Dt = Dm is known (i.e., 23.82 mm here). When Dt = Dm, then p = 1/q and, therefore, p = 1/[0.01306(23.82–1.82)] = 3.48.

### Glaucoma conversion

The primary outcome event for NTG suspects was conversion to abnormal glaucomatous VF or presence of a new localized RNFL defect in superotemporal or inferotemporal regions on disc/retinal photographs. Abnormal glaucomatous VF was defined when the pattern deviation plot became abnormal (presence of a cluster of ≥ 3 non-edge points on the pattern deviation plot, with a probability of occurring in < 5% of the normal population, and with one of these points having the probability of occurring in < 1% of the normal population), with a pattern standard deviation (PSD) having a *P*-value < 0.05 on two consecutive tests, as confirmed by two glaucoma specialists (H.Y.P. and C.K.P.). VF conversion was confirmed after the clinical investigation, and whether the VF change was likely due to glaucoma or other, confounding conditions, such as cataract, macular disease, or other non-glaucomatous conditions was determined. A localized RNFL defect was defined as a dark, wedge-shaped area, the tip of which was in contact with the optic disc border, which had a bright, striated appearance than the surrounding RNFL on red-free fundus photography. The presence of a new RNFL defect was defined as a newly developed localized RNFL defect in an area where there had previously been no defect, confirmed by two glaucoma specialists (H.Y.P. and C.K.P.)^3,56^. These criteria of glaucoma conversion were included because serial RNFL examinations based on red-free fundus photography are known to be more sensitive than other methods for detecting progression of glaucoma, and localized RNFL changes are frequently observed in NTG eyes. In addition, all NTG suspects were naïve to treatment at baseline and subjects were censored when starting medication due to NTG conversion.

### Statistical analysis

The interobserver reproducibility of the ONH parameter measurements was evaluated; two observers (D.Y.S. and S.J.J.) performed measurements in 30 randomly selected eyes, and intraclass correlation coefficients (ICC) and 95% confidence intervals (CIs) were calculated. According to Fleiss^57^, ICCs ≥ 0.75, 0.40–0.75, and ≤ 0.4 are considered to be excellent, moderate, and poor, respectively. We used Student’s *t * test to compare continuous variables, and the chi-square test to compare categorical variables. Hazard ratios (HRs) for the associations between baseline risk factors and conversion to glaucoma were obtained by Cox regression analysis, with glaucoma conversion as the dependent variable; the following were the independent variables: age at diagnosis, gender, family history of glaucoma, medication of DM and/or systemic hypertension, history of migraine, best-corrected visual acuity, axial length, central corneal thickness, mean baseline IOP, mean IOP during follow-up, baseline MD and PSD of VF, baseline average peripapillary RNFL thickness, baseline average macular GC/IPL thickness, presence of DH, disc area, linear cup-to-disc ratio, disc tilt ratio, disc torsion degree, disc–foveal angle, PPA area, prelaminar thickness, and group (classified according to LC thickness and LC depth-PPS). Cut-off values for LC thickness and LC depth were determined by logistic regression analysis, and eyes were categorized into groups using the calculated cut-off values (180.5 μm for LC thickness and 424.0 μm for LC depth). Independent variables yielding *P-*values < 0.10 in the univariate model were included in the multivariate model. *P*-values < 0.05 were considered statistically significant. For categorical covariates, Kaplan–Meier survival curves were generated, and the log rank test was used to compare risk among groups. Analyses were performed on the total cohort, and in each group (divided into eyes with myopia [axial length ≥ 24.0 mm] and without myopia). All statistical analyses were performed using SPSS for Windows software (ver. 16.0; SPSS Inc., Chicago, IL, USA).

### Consent to participate

Inform consent was obtained from patients for publication of scanned images of retina in an online open-access publication.
